# Care and outcome of a child with erythema multiforme complicated by secondary bacterial infection: a case report

**DOI:** 10.3389/fped.2026.1916599

**Published:** 2026-10-26

**Authors:** Nannan Li, Zhe Zhang, Yujie Lu, Huanling Wu, Yu Zhang, Zhong Li, Fenghui Cong

**Affiliations:** 1 Hematology-Oncology Department, Dalian Women and Children’s Medical Group, Dalian, Liaoning, China; 2 Department of Pharmacy, Dalian Women and Children’s Medical Group, Dalian, Liaoning, China; 3 Department of Nursing, Dalian Women and Children’s Medical Group, Dalian, Liaoning, China

**Keywords:** child, erythema multiforme, multistep nursing, oral chemical injury, *staphylococcus aureus*

## Abstract

Erythema multiforme (EM) is an acute mucocutaneous disorder usually triggered by infections. Chemical injury with secondary bacterial infection is a rare trigger in children. We report an 11-year-old boy who initially had a punctate oral ulcer, in whom overnight buccal metronidazole likely caused additional chemical irritation, followed by secondary *Staphylococcus aureus* infection and a subsequent diagnosis of EM. Laboratory findings revealed a marked decrease in CD4+ T cells (185/*μ*L) and a reduced CD4/CD8 ratio (0.66). Lesions progressed from the oral cavity to the extremities, with severe lip and oral involvement. Initial burn-based wound care was ineffective. Following diagnosis, a structured wound care protocol was implemented, consisting of sequential topical ethacridine lactate, mupirocin, compound triamcinolone acetonide, and recombinant human epidermal growth factor, with continuous low-flow oxygen. The lesions improved markedly, lip ulcers healed, oral function returned, and the patient was discharged well. This case underscores the need for early EM recognition in children with atypical oral lesions and describes a structured, multistep nursing strategy that was used in this case.

## 1 Introduction

Erythema multiforme (EM) is a rare acute inflammatory disease that can involve the skin, mucous membranes, or both, and it often recurs (1, 2). The peak age of onset is 20 to 40 years, but approximately 20% of cases occur in children (3, 4). Based on clinical manifestations, erythema multiforme is mainly divided into minor and major forms. The minor form typically involves only one mucosa and may be accompanied by symmetrically distributed iris shaped skin lesions (with a concentric ring like appearance) (5). The major form involves two or more mucous membranes with more extensive skin involvement (5). Approximately 70% of patients with either the minor or major form have oral lesions, which may appear in isolation or precede lesions at other sites, presenting as diffuse erosions, blisters, and ulcers of the oral mucosa (6). Intraoral lesions are mostly located on the non-keratinized mucosa of the anterior part of the mouth, and the lips often show cracking, bleeding, swelling, and crusting, but heal without scarring (6–9). The typical skin lesions of this disease are caused by dermal inflammation mediated by CD4^+^ T lymphocytes and monocytes, while erosions and ulcers result from a T cell mediated immune reaction against antigen expressing keratinocytes, leading to apoptosis and vesicle formation (7).

Various factors can trigger erythema multiforme, including infections (viral or bacterial), drugs, and neoplastic diseases, with infection being particularly common (10). Because the disease is highly variable, especially when confined to the oral cavity, it can be easily confused with other oral mucosal diseases such as herpetic stomatitis, aphthous ulcers, and drug allergic reactions, leading to misdiagnosis or delayed diagnosis.

We report a case of an 11-year-old child who initially presented with a punctate oral ulcer. Overnight buccal administration of metronidazole likely caused additional chemical irritation of the oral mucosa, followed by secondary *Staphylococcus aureus* infection, and was subsequently diagnosed with oral EM. Through the nursing review of this case, we aim to summarize the nursing experience of pediatric oral erythema multiforme, including key points such as condition observation, oral and skin mucosal care, infection control, and symptomatic supportive care, in order to provide a reference for clinical nurses managing such immune mediated oral diseases.

## 2 Patient presentation

The patient was an 11-year-old boy with no personal or family history of immunodeficiency. A detailed clinical timeline is provided (Figure 1). Three days before admission, he developed a punctate ulcer around a molar without an obvious trigger. His parents had him take two tablets of metronidazole buccally at the ulcer site overnight and also administered oral Houttuynia cordata (a traditional Chinese herbal medicine with anti-inflammatory and antiviral properties). After this treatment, the boy developed lip ulcers that gradually increased in size. The day before admission, he visited the oral clinic of our hospital and was treated with topical mouthwash and Kaihoujian Aerosol (a Chinese patent medicine containing extracts of Ardisia crispa, Sophora tonkinensis, cicada slough, and menthol, used for oral and pharyngeal inflammation). He was admitted because his lip ulcers did not improve and fever developed (Figure 2). The initial diagnosis was suspected chemical burn of the oral mucosa. Initial treatment included vitamin C injection (100 mg/kg, qd) for symptomatic relief, along with topical oral inflammatory spray and Kaihoujian Aerosol, as well as ibuprofen suspension for fever reduction. Initial nursing care for the lip crusts followed the burn care protocol twice daily. The specific steps included debridement and eschar removal, disinfection with povidone iodine, and topical application of mupirocin ointment combined with moist burn ointment (MEBO). The entire process was accompanied by low flow oxygen to assist wound healing. Pain intensity, oral intake, hydration status, and functional recovery were assessed and documented daily as part of routine nursing care. Nutritional status was evaluated by monitoring food intake and body weight. After admission, the condition progressively worsened. The oral and lip ulcers increased with purulent secretions and halitosis, large ulcers appeared in the bilateral retromolar areas and buccal mucosa, and bullae and ruptured lesions appeared on the extremities (Figure 3).

**Figure 1:**
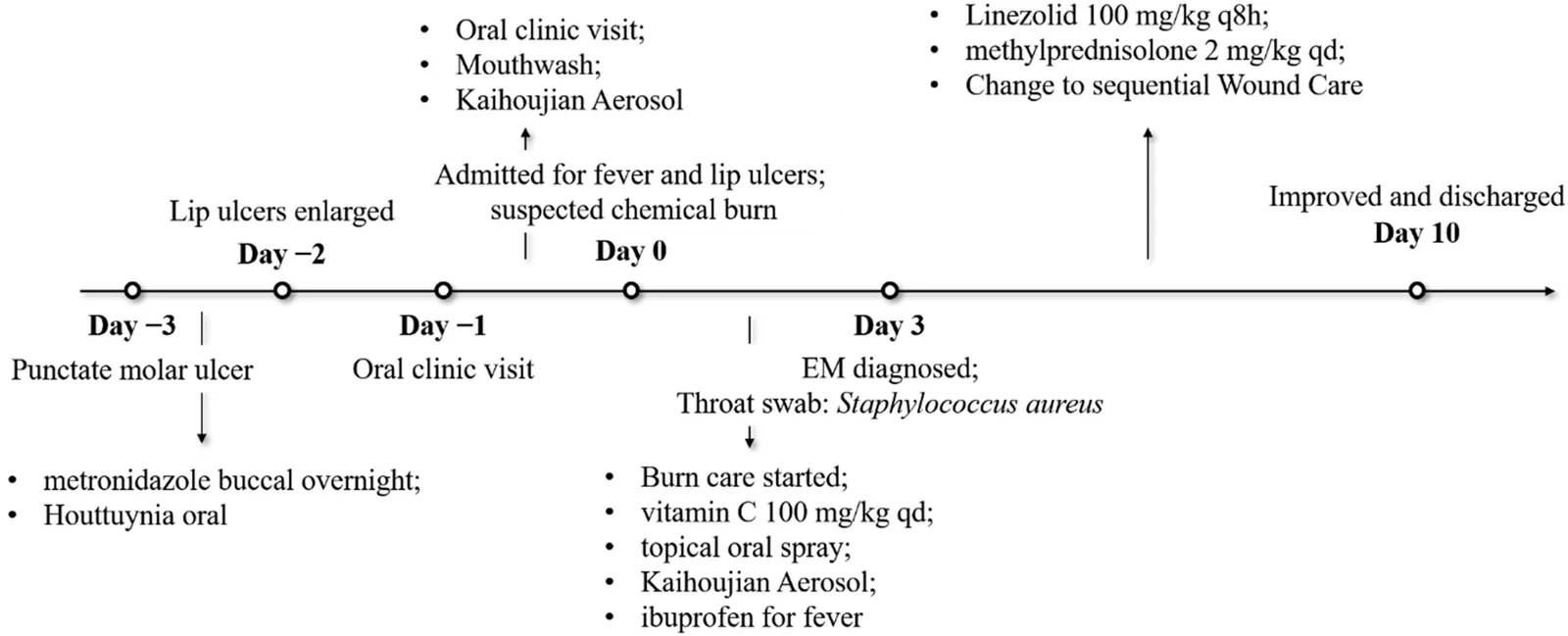
Clinical timeline of the patient from initial oral ulcer to discharge, showing key diagnostic, therapeutic, and nursing events. Day 0 indicated the day of admission.

**Figure 2:**
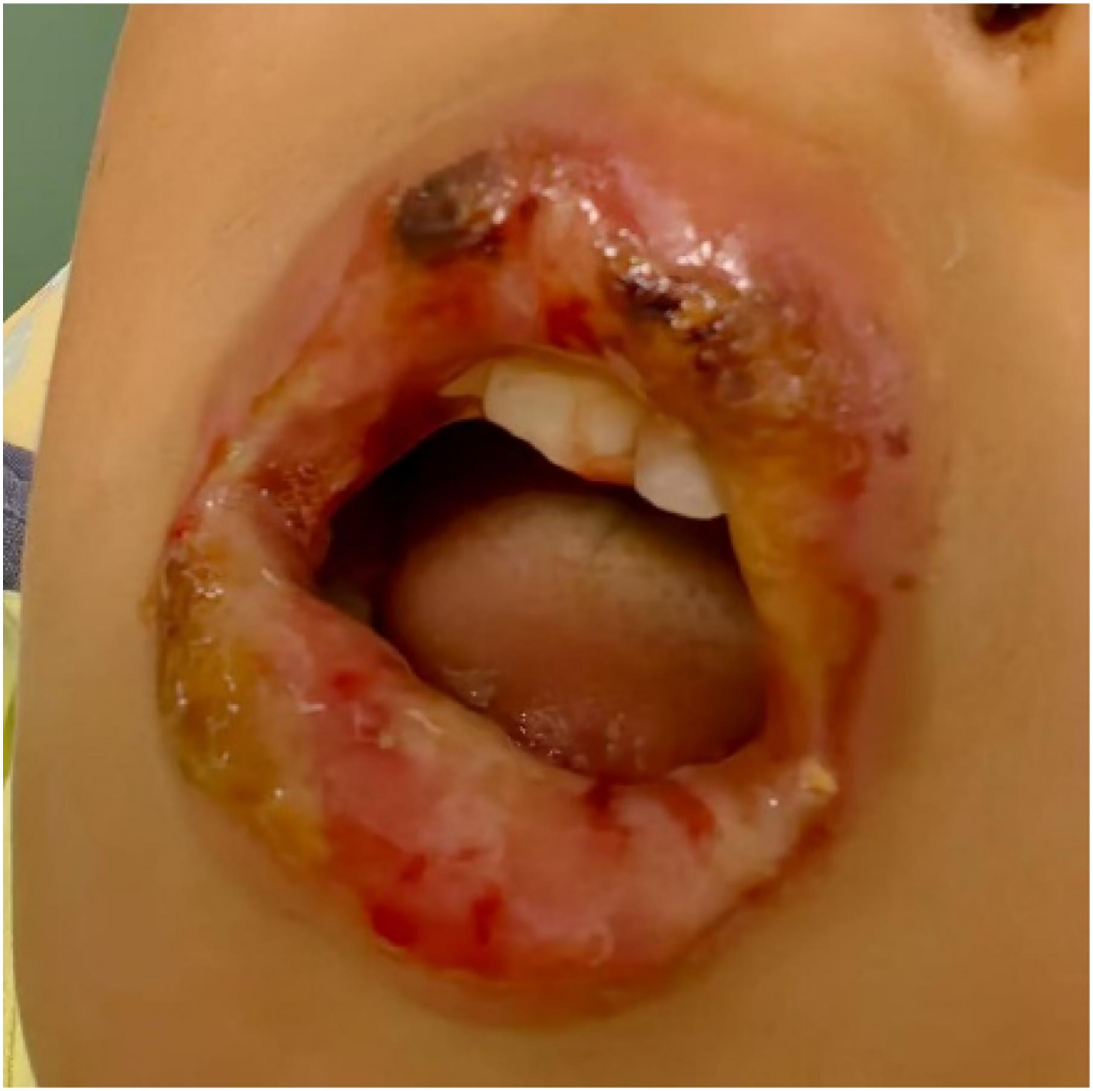
Initial presentation of the lips and oral mucosa. Edematous lips with extensive ulceration, hemorrhagic crusting, and purulent exudate.

**Figure 3:**
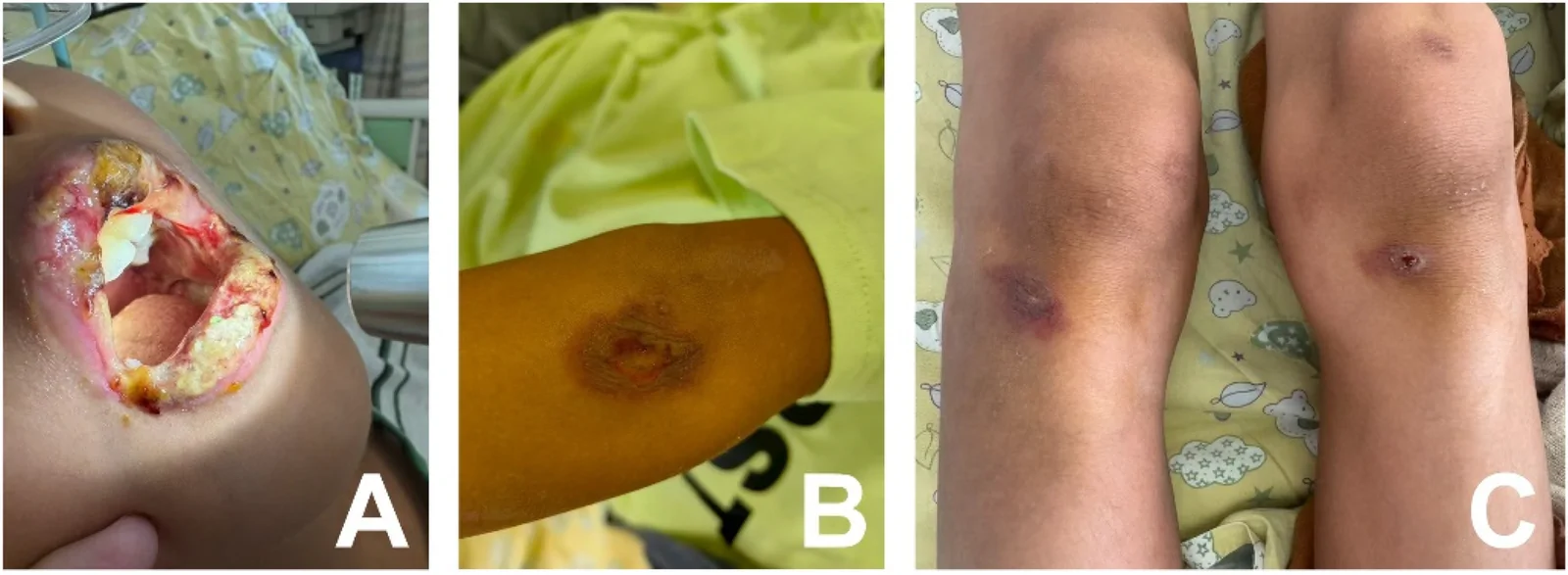
Disease progression during hospitalization. **(A)** Deterioration of lip lesions with marked swelling and diffuse yellowish purulent exudate covering the entire lips, with no intact mucosal areas visible. **(B,C)** Characteristic EM lesions on the arm and leg.

On day 3 of hospitalization, throat swab culture grew *Staphylococcus aureus*. Lymphocyte subset analysis showed abnormal findings, primarily a marked decrease in CD4+ T cells (185/μL) and a reduced CD4/CD8 ratio (0.66). Cytokine testing also showed abnormal values with IL-6 at 23.17 pg/mL and IL-10 at 11.02 pg/mL. herpes simplex virus IgM (HSV-M) was negative [<0.500 signal-to-cutoff ratio (S/Co)], and respiratory *Mycoplasma* testing was negative (Table 1). Based on these microbiological and immunological findings, the patient was diagnosed with Erythema multiforme (EM), and secondary *S. aureus* infection was identified. The diagnosis of EM was based on the acute onset of mucocutaneous lesions, the presence of tense bullae on the extremities, and severe oral mucosal involvement with hemorrhagic lip crusting. No classic targetoid lesions were observed. Dermatology and oral medicine consultations were obtained, and both supported the diagnosis of EM. Skin or mucosal biopsy was considered but not performed because the parents declined the procedure and the clinical presentation was considered sufficiently characteristic. The differential diagnosis included Stevens-Johnson syndrome/toxic epidermal necrolysis (SJS/TEN), primary herpetic gingivostomatitis, Mycoplasma-induced mucositis, drug-related mucosal injury, and chemical burn. The key features supporting or refuting each diagnosis are summarized (Table 2). The treatment was changed to linezolid (10 mg/kg, q8 h) for anti-infection and methylprednisolone sodium succinate (2 mg/kg, qd) for anti-inflammation. MEBO was immediately discontinued, and a sequential wound care protocol was initiated three times daily, including oral rinse, debridement, ethacridine lactate wet compress, mupirocin, and alternating triamcinolone acetonide and recombinant human epidermal growth factor with low-flow oxygen (Table 3).

**Table 1:** The clinical information of the pediatric patient.

Patient Presentation	Case	Reference Range
Routine Blood Test Parameters		
WBC (×10^9^/L)	6.64	4.60–11.90
NEUT% (%)	62.8	32.0–71.0
LYMPH (%)	20.5	22.0–57.0
MON (%)	15.5	3.0–12.0
CRP (mg/L)	10.53	0.00–10.00
Immune and Cytokine Parameters		
IL-6 (pg/mL)	23.17	0–16.6
IL-10 (pg/mL)	11.02	0–4.9
lymphocyte subset parameters		
CD3 (%)	46.29	57.10–73.43
CD4 (%)	17.06	24.00–38.72
CD8 (%)	25.90	21.01–33.94
CD56 (%)	30.16	10.01–26.98
CD19 (%)	24.64	9.19–19.48
CD4/CD8	0.66	0.81–1.66
Lymphocyte count	1,129	2,020–3,610
Total CD3⁺ T-cell count	523	1,325–2,276
CD4⁺ T-cell count	185	531–1,110
CD8⁺ T-cell count	282	480–1,112
Pathogen-specific parameters		
HSV-M (S/Co)	<0.500 (negative)	0.00–0.90
MP-DNA	negative	negative

WBC, white blood cell; NEUT%, neutrophil percentage; LYMPH, lymphocyte; MON, monocyte; CRP, C-reactive protein; IL, interleukin; CD, cluster of differentiation; HSV-M, herpes simplex virus IgM; S/Co, signal-to-cutoff ratio; MP-DNA, Mycoplasma pneumoniae DNA.

**Table 2:** Differential diagnosis of EM in this case.

Condition	Supportive features	Features against/exclusion basis
EM	Acute onset; severe oral involvement; extremity bullae; hemorrhagic lip crusting	\
SJS/TEN	Fever; mucosal involvement; bullae	No extensive epidermal detachment; negative Nikolsky sign; no ocular/genital symptoms or signs BSA <10%
Primary herpetic gingivostomatitis	Oral ulcers; fever	No gingival erythema; no typical vesicles; HSV-M negative
Mycoplasma-induced mucositis	Mucositis; fever	No respiratory symptoms; respiratory Mycoplasma testing negative
Drug-related mucosal injury	History of drug use	Topical metronidazole; progression after discontinuation; no eosinophilia or systemic rash
Chemical burn	Chemical exposure	Lesions beyond contact site; distant extremity bullae; poor response to burn care

EM, erythema multiforme; SJS/TEN, Stevens-Johnson syndrome/toxic epidermal necrolysis; BSA, body surface area; HSV-M, herpes simplex virus IgM.

**Table 3:** Sequential nursing protocol for oral and cutaneous lesions (single session).

Step	Agent/Intervention	Concentration/Dose	Frequency	Duration	Administration method	Clinical rationale
1	Chlorhexidine mouthwash	0.12%, 5 mL	Once per session	30 s- 60 s	Oral rinse	Reduce oral bacterial load
2	Debridement and eschar removal	\	\	\	Sterile procedure	Remove necrotic tissue
3	Ethacridine lactate solution	0.1%, 10 mL	Once per session	10 min	Wet compress	Broad-spectrum antibacterial
4	Mupirocin ointment	2%, approx.0.1 g	Once per session	1 h	Apply (thin layer)	Inhibit *S. aureus*
5a	Compound triamcinolone acetonide	0.1%, approx.0.1 g	Once per session (alternating with 5b)	30 min	Apply (thin layer)	Suppress local inflammation
5b	Recombinant human epidermal growth factor	20 μg/g, approx. 0.1 g	Once per session (alternating with 5a)	10 min	Apply (thin layer)	Promote epithelial regeneration
6	Low-flow oxygen	3 L/min	Once per session (only during 5b)	Until dry, approx. 10 min	Local insufflation	Improve wound microenvironment

S aureus, Staphylococcus aureus.

On day 5, culture of the lower extremity blister fluid showed mixed bacterial growth with *S. aureus* as the dominant organism. Antimicrobial susceptibility testing showed that the isolate was susceptible to linezolid. Thereafter, the extremity blisters gradually improved. The right arm lesion became crusted, and the bilateral knee pustules ruptured and the crusts fell off. On day 9, blood culture was negative. On day 10, the right arm wound was well crusted, both knees were crusted, and the lip ulcers were markedly reduced without purulent secretions. Some crusts had fallen off, and only a few ulcers remained on the bilateral buccal mucosa and upper gingiva (Figure 4). Before discharge, routine blood tests showed normal results. The patient recovered his appetite and spirit, was afebrile, and met the criteria for discharge. Discharge instructions included continued local care and outpatient follow up.

**Figure 4:**
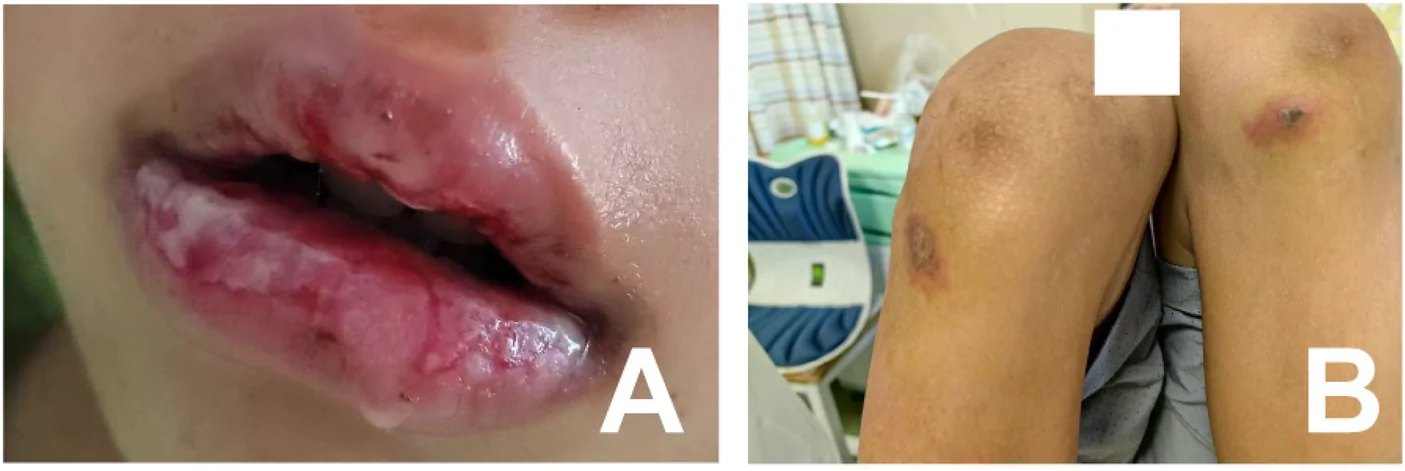
Clinical improvement after treatment. **(A)** Lip ulcers are markedly reduced, without purulent secretions. Some crusts have detached, and only a few small residual ulcers remain. **(B)** Erythema multiforme lesion on the knee showing central crusting with persistent surrounding erythema.

## 3 Discussion

Erythema multiforme (EM) is an autoimmune mucocutaneous disorder with acute onset and typically no prodromal symptoms (11). It is classified into EM minor without mucosal involvement and EM major with mucosal involvement (11). The pathogenesis involves a type IV hypersensitivity reaction to infectious triggers (such as herpes simplex virus types 1 and 2, Mycoplasma pneumoniae) or drugs (such as nonsteroidal anti-inflammatory drugs, antiepileptics, and certain antibiotics, particularly penicillins and sulfonamides) (12). Oral manifestations occur in approximately 70% of patients (7), initially presenting as edematous macules on the lips and buccal mucosa, which progress to multiple vesicles and bullae that rupture to form pseudomembranes. Some patients experience recurrent episodes, and these cases often respond poorly to immunosuppressive therapy (7).

EM traditionally occurs predominantly in young adults, but up to 20% of cases develop in children, and lesions may be limited to the oral cavity, a fact that may be underrecognized by pediatric dentists. We report an 11-year-old boy with EM whose trigger differed from the common causes and involved a more complex process. The patient initially had a punctate oral ulcer. Overnight buccal administration of metronidazole may have aggravated the mucosal injury. Disruption of the mucosal barrier facilitated secondary *Staphylococcus aureus* infection, which was identified in association with both oral and skin lesions. The patient presented with acute oral ulcers. Because the early manifestations were atypical and mimicked acute stomatitis, and were attributed to the chemical injury from metronidazole, EM was not initially considered in the differential diagnosis. The diagnosis was confirmed only after the sequential appearance of bullae, vesicles, and pustules on the extremities during hospitalization and the isolation of *S. aureus*. Therefore, although EM is relatively uncommon, it should always be considered in the differential diagnosis of acute-onset oral inflammatory lesions in children, even when the presentation is atypical.

The mucocutaneous lesions of EM are essentially T cell-mediated immune responses (13). Early lesions show a predominant infiltration of CD4⁺ T cells, while in the blistering stage, CD8⁺ cytotoxic T cells become the dominant population and cause epidermal separation by directly damaging keratinocytes (14). In infection-induced EM, a decrease in peripheral blood CD4⁺ T cell counts and a reduced CD4/CD8 ratio are frequently observed (14). Two mechanisms may explain this observation. Activated T cells migrate to the skin and mucosa, leading to their transient depletion in the circulation. In addition, superantigens produced by certain pathogens activate T cells non-specifically, which triggers activation-induced cell death and causes a marked reduction in CD4⁺ T cells (14–16). The peripheral blood findings in our patient were consistent with this pattern: CD4⁺ T cells were markedly reduced to 185/μL, the CD4/CD8 ratio was inverted at 0.66, and IL-6 and IL-10 levels were elevated. These findings may reflect immune dysregulation. *S. aureus* may produce superantigens that could contribute to this process. The peripheral blood findings in our patient included a marked reduction in CD4+ T cells to 185/μL, an inverted CD4/CD8 ratio of 0.66, and elevated IL-6 and IL-10 levels. These findings were observed at a single time point and are not specific to EM. They may suggest immune dysregulation, but they do not establish a specific immunological mechanism. *S. aureus* may produce superantigens that could contribute to this process, although superantigens were not directly measured in this case.

Oral mucosal lesions in EM are often intensely painful and can reduce oral intake. Severe cases may require hospitalization for intravenous fluids and electrolyte replacement (1, 12). Current local treatment for mucosal involvement is largely based on expert opinion and small case series, and no controlled studies have established the superiority of any specific regimen. Commonly used measures include high-potency topical corticosteroid gels and oral rinses containing antiseptic or anesthetic solutions (17). However, when the oral mucosa is extensively eroded, crusted, and secondarily infected, symptomatic treatment alone is often insufficient to control disease progression (7, 12). Our nursing experience with this case suggests that severe oral EM with extensive mucosal lesions may require a structured local wound care strategy. The initial burn-care protocol proved ineffective for this lesion. The nursing protocol was therefore changed to a sequential approach. The sequential wound care protocol used in this case was developed based on our institutional experience rather than a pre-existing published regimen. The sequence was designed as follows. Initial cleansing and debridement were performed to remove necrotic tissue and reduce bacterial load. An antiseptic wet compress was then applied, followed by a topical antimicrobial agent. Finally, anti-inflammatory and regenerative agents were used alternately, with low-flow oxygen applied only during the regenerative step. This stepwise sequence aimed to address infection, inflammation, and tissue repair in a structured manner (18–22). The timing of these agents was guided by clinical signs of infection control, including reduction of purulent discharge, decreased halitosis, and improvement of the wound base. A topical corticosteroid (compound triamcinolone acetonide) was added to the routine wound care to directly target the local T cell-mediated inflammation (18). The protocol also combined mupirocin ointment for targeted elimination of *S. aureus*, ethacridine lactate solution for broad-spectrum antibacterial coverage, and recombinant human epidermal growth factor to promote epithelial regeneration (19–21). Recombinant human epidermal growth factor has shown benefit in chemotherapy- or radiotherapy-induced oral mucositis. In a randomized trial of erosive-ulcerative oral mucosal disease, adding recombinant human epidermal growth factor to triamcinolone/lidocaine significantly shortened mucosal healing time and pain duration compared with triamcinolone/lidocaine alone (23). However, the MASCC/ISOO 2020 mucositis guidelines do not currently grade topical corticosteroids or EGF for oral mucositis (24). Our protocol combined these agents based on institutional experience, and further studies are needed to validate this approach. Continuous low-flow oxygen insufflation was applied throughout the procedure as an adjunctive measure based on local clinical practice, with reference to reported potential benefits on the wound microenvironment and wound healing (22). This integrated approach addressed the underlying infection, the inflammatory process, and the need for tissue repair.

This case suggests that for children with acute-onset oral inflammatory lesions, even when the trigger appears to be chemical or physical, EM should remain in the differential diagnosis if the condition progresses and bullous skin lesions develop. Early recognition can shorten the diagnostic delay and facilitate timely adjustment of treatment and nursing care. Regarding wound management, the initial burn-care protocol proved ineffective in this case. It was subsequently replaced by a sequential nursing strategy that simultaneously addressed infection, inflammation, and tissue repair. This shift indicates that local management of immune-mediated oral mucosal lesions cannot be simply equated with that of ordinary infectious or traumatic wounds. An appropriate local wound care strategy may support mucosal healing. This experience is based on a single case and requires further clinical validation, but it may serve as a practical reference for the oral wound management of childhood EM in similar complex situations. Ophthalmological examination was not performed because the patient had no ocular symptoms, which is a limitation of this report. Nevertheless, this case may serve as a practical reference for the oral wound management of childhood EM in similar complex situations.

## 4 Conclusion

In conclusion, we report an 11-year-old boy who developed erythema multiforme following oral chemical injury from overnight buccal metronidazole, with secondary *Staphylococcus aureus* infection identified. Initially misdiagnosed as a chemical burn, the correct diagnosis was established after bullous lesions appeared on the extremities. The patient recovered after a sequential wound care protocol. This case highlights the importance of considering erythema multiforme in children with acute oral inflammatory lesions to avoid diagnostic delay. When conventional wound care fails, a structured local wound care strategy may be considered to support mucosal healing. This experience provides a nursing reference for similar rare cases.

## Data Availability

The original contributions presented in the study are included in the article/Supplementary Material, further inquiries can be directed to the corresponding author.
